# Characterization of an Indigenous Lytic Phage Targeting Multidrug-Resistant *Salmonella enterica*

**DOI:** 10.1080/29933935.2025.2452485

**Published:** 2025-01-21

**Authors:** Ebele Onuigbo, Paul Akpa, Anthony Attama, Stephen Emencheta, Emmanuel Eze, Chinonye Obeta

**Affiliations:** a Department of Pharmaceutical Microbiology and Biotechnology, Faculty of Pharmaceutical Sciences, University of Nigeria, Nsukka, Nigeria; b Department of Pharmaceutics, Faculty of Pharmaceutical Sciences, University of Nigeria, Nsukka, Nigeria

**Keywords:** Bacteriophage, *Salmonella enterica*, lytic, native, therapy

## Abstract

Multidrug-resistant pathogens have prompted the use of lytic bacteriophages. An indigenous novel lytic bacteriophage against *Salmonella enterica* strains from environmental wastewater was isolated and characterized using phage survivability study, adsorption curve, one-step curve, optimal multiplicity of infection, and phage-killing assay. The *Salmonella* strains CP90 and CP23 isolated from the same source were biochemically and molecularly characterized. The *Salmonella* strains CP90 and CP23 had 96.24% and 97.18% pairwise identity respectively with *S. enterica*. Both were resistant to B-lactam Aminoglycosides, Penicillin, and Phenicol class of antibiotics. The phage performed better in an alkaline medium and below 50°C. About 80% of the phage had an adsorption rate of 12 min and a latent period of 20 min. About 55 PFU/cell of the phage was released during a single replication cycle, inhibiting bacteria growth for up to 5 h. The characterization of this indigenous phage suggests its therapeutic potential against multidrug-resistant *Salmonella* species.

## Introduction

Salmonellosis is an infection caused by *Salmonella* species with an annual burden of 21.6 million invasive and 93.8 million noninvasive illnesses worldwide, resulting in about 433,000 and 155,000 deaths respectively.^1^ Its susceptible hosts are humans, domesticated animals, and wildlife^2^ with poultry birds holding the highest place of contamination. About 20% of poultry products worldwide are contaminated with *Salmonella* species.^3,4^ The organism is extensively distributed worldwide^5,6^ and has two species; *S. enterica* and *S. bongori* based on the acquisition of *Salmonella* pathogenic islands 1 and 2. Most species survive in environments like frozen water, soil, water bodies, plants, and fecal matter.^7^ *S. enterica* causes invasive and noninvasive salmonellosis and is responsible for about 99% of all *Salmonellae* infections.^8,9^ The effective control of salmonellosis involves techniques like disinfection, pasteurization, irradiation, high hydrostatic pressure, antibiotics, and biocides application.^10,11^ However, the adverse effects caused by these methods on man, animals, crops, food, and the environment have raised concerns.^10,12,13^ In food processing, an undesirable change in the nutritional and sensory qualities of food has been reported.^13^ Sadly also, the frequent abuse of conventional antibiotics in the control of salmonellosis has led to the evolution and transmission of multidrug-resistant strains of *Salmonella*. There is evidence of antimicrobial resistance of *Salmonella* species to first-line drugs like chloramphenicol, ampicillin, trimethoprim-sulfamethoxazole, and now fluoroquinolones.^14–17^ Multidrug-resistant (MDR) *Salmonella* strains are responsible for at least 100,000 salmonellosis and *Salmonella* outbreaks yearly.^18,19^ Thus, they are classified by the Centers for Disease Control and Prevention (CDC) as a serious threat to human health.^18^

With increasing health challenges associated with antimicrobial resistance and the undesirable effects of physical and chemical sterilants, bacteriophages can serve as a new norm for the control of bacterial pathogens. Phages are viruses and parasites of bacteria that multiply only within the host. They are mostly described as lytic or lysogenic although other forms exist. A lysogenic (temperate) phage after the synthesis of its genetic material integrates its genome into the host chromosome and multiplies alongside the host cell. The progeny of the lytic (virulence) phage accumulates within the cytosol of the host and secretes lytic enzyme (endolysin), ultimately lysing the cell wall of the bacteria. Bacteriophages are considered safe for the following reasons: they are immuno-tolerant with little or no adverse effects,^20^ produces little or no change as a food additive,^12^ replicates easily with low-cost production, co-administration with antibiotics for effective treatment of resistant pathogens, easier genetic manipulation to improve host range of susceptible bacteria.^21,22^

*Salmonella* phages have been isolated and characterized from diverse environments.^11^ *Salmonella* phage exploitation in various fields has also been documented, including *Salmonella* phage typing for epidemiological purposes, phage biosensor for detection of specific *Salmonella* species in food and clinical laboratories, control of bacterial infection in crops and agricultural products, and as prophylaxis for livestock breeding.^23–28^ Despite the number of *Salmonella* phages being reported and tested, the current phage resource library is still limited compared with the number of resistant *Salmonella*.^11,29,30^ Also, there are huge lapses of phage studies in Africa and no known phage trial is ongoing in Nigeria^31^ despite being the most dominant entity influencing our ecosystem. As some diseases are domicile in some regions, the cure will be most effective if managed within the same region, therefore, phages serve as a better therapeutic option, being environmentally friendly and ubiquitously available. Control of salmonellosis in ready-to-eat food, livestock farming, crop farming, and environmental waste management can also be achieved in Africa using phages.

Thus, this investigation aims to characterize lytic bacteriophages from environmental wastewater samples using isolated bacteria from the same source as the host system.

## Materials and methods

### Reagents

Gram stain and biochemical reagents, Luria Broth (Titan Biotech, India), SM buffer (50 mm Tris-HCl, pH 7.5, 100 mm NaCl, 8.0 mm MgSO_4_, and 0.01% gelatin), phosphate-buffered saline (Lab M, Heywood, UK), calcium chloride (Merck, USA), 100 ml of environmental wastewater samples in Nsukka, Enugu State, Nigeria were pooled from different sites including fishery, piggery, swimming pool, UNN central sewage, abattoir, and poultry.

### *Isolation of* salmonella

Pre-enrichment medium using 9 mL of buffered peptone water (Lab M, Heywood, UK) and 1 mL of the environmental wastewater samples was prepared and incubated overnight at 37°C. For the selective enrichment, 0.1 mL of the pre-enrichment medium was inoculated in 10 mL of Rappaport-Vassiliadis broth (Oxoid, Hampshire, UK), and incubated at 41.5°C for 24 h. A loopful (ca. 10 µL) from Rappaport- Vassiliadis broth was inoculated onto the surface of Xylose-Lysine-Deoxycholate, (XLD) agar, (Oxoid, UK) or Salmonella-Shigella (SSA) agar (Chaitanya AgroBiotech, India) and subsequently incubated at 37°C for 24 h. The presumptive *Salmonella* isolates were further sub-cultured on XLD or SSA to obtain pure colonies of the isolates. The Gram staining was done, and the isolates were further characterized biochemically using the Triple Sugar Iron Test (TSI), Catalase test, Urease test, SIM (Sulfur, Indole. Motility) agar, and Citrate test with controls added where relevant.^32^

### Genomic DNA extraction and electrophoresis

The nutrient broths containing the host bacteria (CP90 and CP23) were washed with 100 µL of Ringers solution and centrifuged for 10 min at 10,000 rpm. DNA extraction and molecular characterization using electrophoresis and 16S rRNA gene amplification were performed according to ZrFungal/Bacterial DNA MiniPrep^TM^ manufacturer’s instructions (Zymo Research, USA).

MiniPrep™ manufacturer’s guide is the protocol developed by Zymo research group for DNA extraction. More information can be found on Zymo Research Database.

To confirm the presence of DNA, a Loading buffer was added to each DNA or PCR sample and allowed to solidify. The agarose gel was placed into the gel box (electrophoresis unit) and filled with 1 × TAE (or TBE) until the gel was covered. A molecular weight ladder was carefully loaded into the first lane of the gel. The gel was run at 80–150 V for about 1–1.5 h after which the electrode was disconnected and the DNA fragment visualized under a UV transilluminator.^33^

### 16S rRNA Gene amplification of bacterial isolates

The PCR mix was made up of 12.5 µL of Taq 2X Master Mix from New England Biolabs (M0270); 1 µL each of 10 µM forward (27F: AGAGTTTGATCMTGGCTCAG) and reverse (1525 R: AAGGAGGTGWTCCARCCGCA) primer; 2 µL of DNA template and then 8.5 µL Nuclease free water. Cycling conditions for the amplification of the 16S rRNA gene were; Initial denaturation at 94°C for 5 min, 36 cycles of denaturation at 94°C for 30 sec, annealing at 56°C for 30 sec, elongation at 72°C for 45 sec, and a final elongation step at 72°C for 7 min and hold temperature at 10°C.^34^

### Sequencing

The 16S rRNA gene was amplified using the PCR and the DNA sequence was screened against the NCBI GenBank to find the most similar identity. The amplified fragments were sequenced using a Genetic Analyzer 3130 × l sequencer from Applied Biosystems using the manufacturers’ manual while the sequencing kit used was that of Big Dye Terminator v3.1 cycle sequencing kit. Bio-Edit software and MEGA X were used for all genetic analyses.

### Antibiotics susceptibility test

Kirby-Bauer disk diffusion method was used to assay the antimicrobial susceptibility profile of the isolates using ten commercially available gram-negative antibiotics multi-disc (Maxicare Medical Laboratory, Nigeria); comprising of Cotrimoxazole (CTM) 30 µg, Chloramphenicol (C) 30 µg, Sparfloxacin (SP) 10 µg, Ofloxacin (OFX) 10 µg, Gentamicin (CN) 30 µg, Ciprofloxacin (CPX) 30 µg, Augmentin (AU) 10 µg, Pefloxacin (PEF) 30 µg, Amoxicillin (AMC) 30 μg, and Streptomycin (S) 30 µg. The test was performed according to the CLSI performance standard for antibiotic susceptibility testing. Briefly, the antibiotic discs were placed on a bacteria lawn of a colony suspension (equivalent of 0.5 McFarland standard) prepared on Mueller-Hinton agar plates and incubated at 37°C for 24 h. The inhibition zones were measured and interpreted accordingly. CLSI breakpoints were used to classify the zone diameter (mm) values as susceptible, intermediate, or resistant.^35^

### Multiple antibiotic resistance index (MARI)

This was done following the procedure of Krumperman, 1983.^36^

(1) $$\frac{\mathrm{The}\;\mathrm{number}\;\mathrm{of}\;\mathrm{antibiotics}\;\mathrm{to}\;\mathrm{which}\;\mathrm{the}\;\mathrm{isolate}\;\mathrm{were}\;\mathrm{resistant}}{\mathrm{The}\;\mathrm{total}\;\mathrm{number}\;\mathrm{of}\;\mathrm{antibiotics}\;\mathrm{against}\;\mathrm{which}\;\mathrm{the}\;\mathrm{isolate}\;\mathrm{was}\;\mathrm{tested}}$$

### Preparation of bacteria lawn

A colony of bacterial culture containing *S. enterica* was suspended in 10 mL of SM buffer. After vortexing, 100 µL of the bacteria suspension (10^5^ CFU/ml) was mixed with 3 mL of molten LB (Titan Biotech, India) and overlaid on Muller-Hinton agar (Titan Biotech, India).^37^

### Phage isolation and enrichment

Using the method of Yuanyuan et al. with a slight modification, 15 mL of each wastewater (from the fish pond, swimming pool, UNN central sewage, abattoir, piggery, and poultry) samples were pooled together into a 100 mL sterile bottle, and vortexed. A 10 mL volume of the cocktail wastewater was centrifuged at 6500 g for 15 min and filtered through a 0.45 µm syringe filter (GE Healthcare Life Sciences, USA). The filtrate was added to 2 mL of nutrient broth containing 100 µL of overnight cultured *Salmonella* and vortexed. The suspension was incubated at 37°C for 48 h. This procedure was repeated.^38^

### Phage enrichment

Briefly, 0.2 mL of 20 mm Calcium Chloride, 6 mL of the phage filtrate, 1.8 mL of 5 × nutrient broth, and 2 mL of an overnight bacterial broth culture were mixed and incubated at 37°C for 48 h. The enrichment medium was then centrifuged at 6500 g for 15 min and the phage filtrate preserved at 4°C.^39^

### Spot assay

A 10 µL volume of the phage filtrate was spotted on the bacteria lawn and incubated overnight at 37°C. The plaques were counted and recorded.^39^

### Double overlay assay (DOA) and phage purification

A 100 µL bacterial isolate, 100 µL phage filtrate and 3 mL of 0.5% soft LB agar were mixed and vortexed. The mixture was overlaid on 17 mL of 1% hard LB agar and incubated overnight at 37°C. Distinct plaques were collected using sterile micropipette tips from the top agar surface and suspended in 10 mL of SM buffer for 2 h at 4°C. After 2 h, the SM buffer was centrifuged at 300 rpm for 30 min and subsequently filtered using 0.45 *μ*m syringe filters.^40^

### Phage count

A 1 mL of phage lysate was serially diluted in SM buffer up to the tenth dilution and a spot assay was prepared with it. Spotting was done in triplicate. Subsequently, to determine the concentration of phages, the highest dilution with the presence of plaques, was used to prepare a double layer agar assay, the number of plaques counted and plaque forming units estimated.^41^

(2) $$\mathrm{PFU}/\mathrm{mL}=\frac{\mathrm{number}\;\mathrm{of}\;\mathrm{plaques}\;\mathrm{formed}\;x\;\mathrm{Reciprocal}\;\mathrm{of}\;\mathrm{the}\;\mathrm{dilution}\;\mathrm{factor}}{\mathrm{Volume}\;\mathrm{of}\;\mathrm{spot}}$$

### Phage survivability studies

The stability of the phage was determined by examining its thermal and pH survivability. Briefly, for pH: About 100 µL of phage lysate was added to 900 µL of LB broth and incubated at 37°C for 1 h. The pH values ranged from 2–13 by adding 1 M HCl or NaOH.

For Temperature: About 100 µL of phage lysate was added to 900 µL of LB broth and incubated at 20, 25, 37, 50, 60, 70, and 80°C for 1 h. Plaque forming units were determined on DOA.^42^

### Adsorption curve

The adsorption rate was performed according to Mulani et al. with a slight modification. The exponentially grown host bacteria (OD_600_ of 0.4) were mixed with the phage lysate at an MOI of 10 and incubated at 37°C for 12 min. A 100 µL volume of the above mixture was collected at 0, 1, 2, 3, 4, 6, 8, 10, and 12 min, and diluted immediately with 900 µL of cold LB broth and centrifuged at 10,000 g for 2 min to remove adsorbed phages. The titer of the unabsorbed phages in the supernatant was then determined on DOA. All experiments were performed in triplicate. The adsorption curve was plotted using the percentage of free phages in the solution over time.^43^

### One-Step growth curve

To determine the one-step growth pattern of the phage, the exponentially grown host bacteria were mixed with the phage lysate at an MOI of 10 and incubated at 37°C for 12 min for adsorption to occur. The infected cells were collected after centrifugation at 10,000 g for 2 min, resuspended into 10 mL nutrient broth and incubated at 37°C. At 10, 20, 30, 40, 50, 60, 70, 80, and 90 min, phage titer was determined on DOA using 100 µL of the phage-infected cells. The plaques formed at each point were counted and used to extrapolate the one-step curve. These experiments were done in triplicate. The latent period and burst size were determined from the graph. Burst size is the ratio of the maximum phage titer to the titer of the original inoculum.^44,45^

### Phage killing assay

A 100 µL of 10^5^ CFU/mL of host bacteria was incubated with 100 µL of phage lysate at different MOI (0.1, 1, 10, 100, and 1000) at 37°C for 12 h. Bacteria growth was monitored by determining the OD_600_ at 1 h intervals. For the negative control, LB mixed with phage was used while LB mixed with bacterial suspension served as the positive control.^29^

### Frequency of appearance of bacterial mutants resistant to the phage

The frequency of appearance of bacterial mutant strains resistant to phage was performed according to Tang et al. with a slight modification. Spot assay technique was performed on the bacteria lawn of host bacteria (CP90 and CP23) as described above. The presence of resistant mutant strains was monitored daily within the lytic zone. About 10 colonies of the observable mutant strains within the lytic zone were collected into 5 ml of nutrient broth and incubated overnight at 37°C. Each of the colonies was serially diluted up to the seventh dilution (10^−7^) using SM buffer. Using a spread rod, the seventh dilution was plated on the nutrient medium by surface dispersion, while 100 µL of the fourth dilution (10^−4^) and 100 µL of phage lysate were used to prepare DOA medium using LB agar.^46^ Following incubation, colony counts were determined and the percentage frequency of the appearance of mutant strains resistant to the phage was determined using the formula:

(3) $$\begin{array}{rl} & \mathrm{Mutant}\;\;\mathrm{strain}\;=\;\left(\mathrm{Average}\;\;\mathrm{colony}\;\;\mathrm{count}\;\;\mathrm{of}\;10^{-4}\mathrm{dilutions}\;\times \;10^{4}\times \;10\right)/\left(\mathrm{Average}\;\;\mathrm{colony}\;\;\mathrm{counts}\;\;\mathrm{of}\;\;10^{-7}\times \;10\right)\;\times 100 \end{array}$$

### Statistical analysis

All data were expressed as the mean ± SD. GraphPad Prism 10.4.0 was used to determine the significant differences applying the Non-Parametric T-test.

## Results

### Bacterial host identification

**Figure 1.**
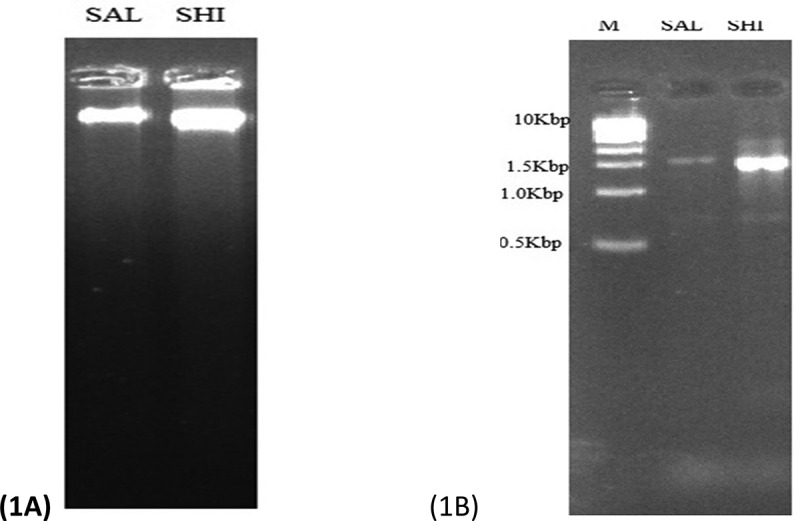
*Salmonella* molecular identification on gel electrophoresis. SAL and SHI represent the CP90 and CP23 respectively. Figure 1A is the gel image of high molecular weight DNA extracted from the isolates and figure 1B is the amplification of the 16S rRNA gene at about 1500bp. M is a 1kbp DNA ladder.

**Figure 2.**
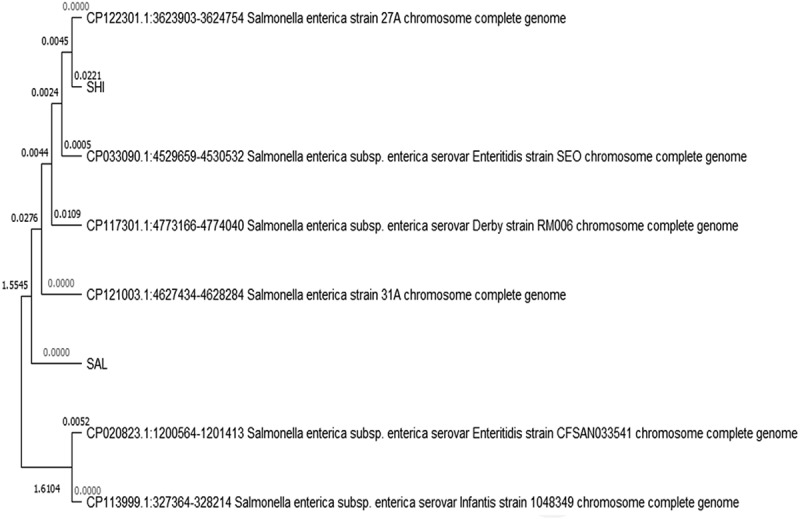
The Phylogenetic tree of the bacteria isolates. From the above structure, the organisms isolated (CP90 and CP23) were Enterobacteriaceae of *Salmonella* origin under *S. enterica*. The *Salmonella* strains CP90 and CP23 had 96.24% and 97.18% pairwise identity with *S. enterica. The 16S rRNA sequence of each isolate was deposited in the* NCBI *GENBANK database with the* accession numbers CP033090 and CP020823 respectively.

Based on the morphological characteristics observed on XLD and SSA media, the host bacteria were typed down to *Salmonella* spp. Host identification was further confirmed using biochemical and molecular characterization. Biochemically, *S. enterica* (CP90 and CP23) were indole and urease negative. Molecularly characterized *E. coli* and *K. pneumonia* were used as the control groups for comparisons. The results of the molecular studies showed that *Salmonella* strains CP90 and CP23 had 96.24% and 97.18% pairwise identity with *S. enterica* as shown in Figures 1, 2. Their 16S rRNA sequence was deposited in the NCBI GENBANK database under the accession numbers CP033090 and CP020823 respectively. The results of the sequence for the isolates are; ACAAGAGGGGAGCGCCCTCCCGAAGGTTAAGCTACCTACTTCTTTTGCAACCCACTCCCATGGTGTGACGGGCGGTGTGTACAAGGCCCGGGAACGTATTCACCGTGGCATTCTGATCCACGATTACTAGCGATTCCGACTTCATGGAGTCGAGTTGCAGACTCCAATCCGGACTACGACGCACTTTATGAGGTCCGCTTGCTCTCGCGAGGTCGCTTCTCTTTGTATGCGCCATTGTAGCACGTGTGTAGCCCTGGTCGTAAGGGCCATGATGACTTGACGTCATCCCCACCTTCCTCCAGTTTATCACTGGCAGTCTCCTTTGAGTTCCCGGCCTAACCGCTGGCAACAAAGGATAAGGGTTGCGCTCGTTGCGGGACTTAACCCAACATTTCACAACACGAGCTGACGACAGCCATGCAGCACCTGTCTCACAGTTCCCGAAGGCACAAATCCATCTCTGGATTCWTMTGTGGATGTCAAGACCAGGTAAGGTTCTTCGCGTTGCATCGAATTAAACCACATGCTCCACCGCTTGTGCGGGCCCCCGTCAATTCATTTGAGTTTTAACCTTGCGGCCGTACTCCCCAGGCGGTCTACTTAACGCGTTAGCTCCGGAAGCCACGCCTCAAGGGCACAACCTCCAAGTAGACATCGTTTACGGCGTGACTACCAGGGWATCTAATCCTGTTTGCTCCMCACGCTTTCGCACCTGAGCGTCAGTCTTTGTCCAGGGGCCGCWTCGCACCGGTATTCCTCCAGATCTCTACGCATTTCACGCTACWCCTGCATCTACCCCCGTCTACCAGACTCAGCCTGCTAGTTCCGATGGCAGGTACCAGCTGAGTCCTGKGAATTCACAGTTYCGAACTTGGACAGAAA for CP90

GSCAAAGGGAAAGGTGTATGSWKRCGTCACGWAGGTTAAGCTACCTACTTCWTTTGCRACCCACTCCCATGGTGTGACGGGCGGTGTGTACAAGGCCCGGGAACGTATTCACCGTGGCATTCTGATCCACGATTACTAGCGATTCCGACTTCATGGAGTCGAGTTGCAGACTCCAATCCGGACTACGACGCACTTTATGAGGTCCGCTTGCTCTCGCGAGGTCGCTTCTCTTTGTATGCGCCATTGTARCACGTGTGTARCCCTGGTCGTAAGGGCCATGATGACTTGACGTCATCCCCACCTTCCTCCAGTTTATCACTGGCAGTCTCCTTTGAGTTCCCGGCCTAACCGCTGGCAACAAAGGATAAGGGTTGCGCTCGTTGCGGGACTTAACCCAACATTTCACAACACGAGCTGACGACAGCCATGCAGCACCTGTCTCACAGTTCCCGAAGGCACAAATCCATCTCTGGATTCWTCTGTGGATGTCAAGACCAGGTAAGGTTCTTCGCGTTGCATCGAATTAAACCACATGCTCCACCGCTTGTGCGGGCCCCCGTCAATTCATTTGAGTTTTAACCTTGCGGCCGTACTCCCCAGGCGGTCTACTTAACGCGTTAGCTCCGGAAGCCACGCCTCAAGGGCACAACCTCCAAGTAGACATCGTTTACGGCGTGCACTACCAGGGWATCTAATCCTGTTTGCTCCCCACGCTTTCGCACCTGAGCGTCAGTCTTTGTCCAGGGGGGCCGCCTTCGCCACCGGTATTCCTCCAGATCTCTACGCATTTCACCGCTACACTGGAAATCTACCCCCTCTACAGACTCAGCTGGCAGTTCGATGCAGTCCAAGATGAGCCTGGGGTATTCACCAGTTCCCCGG for CP23.

### Antibiotic susceptibility test

The results of the Antibiotic Susceptibility Test are shown in (Table 1) with CP90 being susceptible to Sparfloxacin, Ofloxacin, Ciprofloxacin, Streptomycin, Cotrimoxazole, and Pefloxacin but resistant to Gentamicin, Amoxicillin, Augmentin, and Chloramphenicol. CP23 was susceptible to Sparfloxacin, Ciprofloxacin, Ofloxacin, and Cotrimoxazole but resistant to Amoxicillin, Gentamicin, Augmentin, Streptomycin, Chloramphenicol, and Pefloxacin. The MAR1 was 0.4 (CP90) and 0.6 (CP23).

**Table 1.** Antibiotics susceptibility testing of CP90 and CP23.

S/N	Class of Antibiotics	Antibiotics (Concentration)	CP90 (Inhibition zone in mm)		CP23 (Inhibition zone in mm)	
1	Quinolones	Pefloxacin (PEF) 30 µg	25.3 ± 2.4	S	8.00 ± 1.2	R
2		Ofloxacin (OFX) 10 µg	22.7 ± 1.7	S	19.3 ± 1.5	I
3		Ciprofloxacin (CPX) 30 µg	30.3 ± 1.5	S	25.7 ± 1.7	I
4		Sparfloxacin (SP) 10 µg	27.0 ± 1.2	S	24.7 ± 1.7	S
5	B-lactam Combination	Augmentin (AU)10 µg	5.30 ± 1.5	R	0 ± 0.00	R
6	Aminoglycosides	Streptomycin (S) 30 µg	14.0 ± 1.2	I	6.70 ± 1.7	R
7		Gentamicin (CN) 30 µg	9.3 ± 1.5	R	9.30 ± 1.5	R
8	Penicillin	Amoxicillin (AMC) 30 µg	6.70 ± 1.7	R	0 ± 0.00	R
9	Sulfonamides	Cotrimoxazole (CTM) 30 µg	15.3 ± 1.4	I	19.3 ± 1.5	S
10	Phenicol	Chloramphenicol(C) 30 µg	4.70 ± 1.7	R	9.00 ± 1.2	R

The antibiotic susceptibility testing for host bacteria were categorized as susceptible, intermediate and Resistant based on CLSI breakpoint for each antibiotic used against *S. enterica*. The test was done in triplicate and the result represented in standard error of mean.

### Bacteriophage isolation and identification

For the spot assay, clear circular plaques of about 2–3 mm indicating a lytic activity of the spotted bacteriophages were observed on the bacteria lawn containing *S. enterica*. For the DOA technique, clear small plaques were observed and the titer value of the purified phages was 1.3–3.2 × 10^8^ PFU/mL.

### Adsorption and one-step growth curve

**Figure 3.**
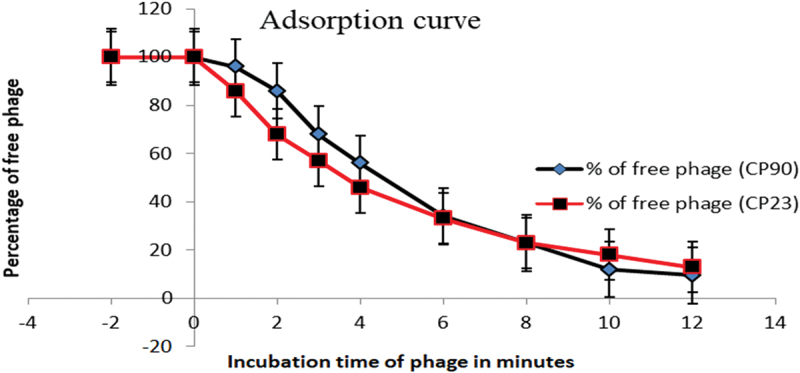
Adsorption curves for CP90 and CP23. The y-axis represents the percentage of free (unabsorbed) phage particles at different intervals. The adsorption curve results show that phage adsorption is about 90% for CP90 and 85% for CP23 within 12 minutes. Each data represents the mean value ± SD of three replicate experiments. At *p* < 0.05, there was a significant difference between the adsorption curve of CP90 and CP23.

**Figure 4.**
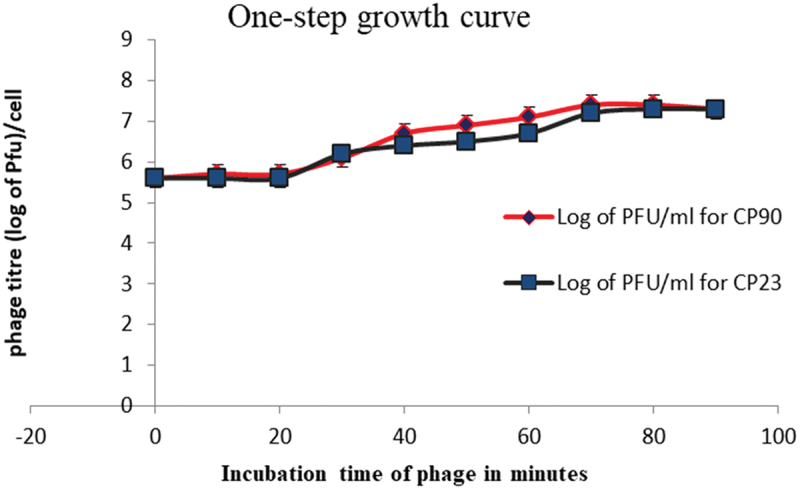
One-step curve of CP90 and CP23. The one-step growth of the lytic phage shows a latent period of 20 min for both CP90 and CP23. The burst size was 70 and 55 Pfu/cell respectively, calculated as the ratio of maximum phage titer after complete lysis to the titer of the original inoculum not absorbed. Each data represents the mean value ± SD of three replicate experiments. At *p* < 0.05, there was a significant difference between the one-step growth curve of CP90 and CP23.

The adsorption profile showed that approximately 90% of the CP90 virions were adsorbed to the *Salmonella enterica* after 12 mins and less than 85% of the CP23 virions were adsorbed to the bacteria host cells after 12 min (Figures 3, 4). The growth pattern showed a latent period of 20 min for both isolates, a burst size of 70 PFU/cell for CP90, and 55 PFU/cell for CP23.

### Phage survivability studies

**Figure 5.**
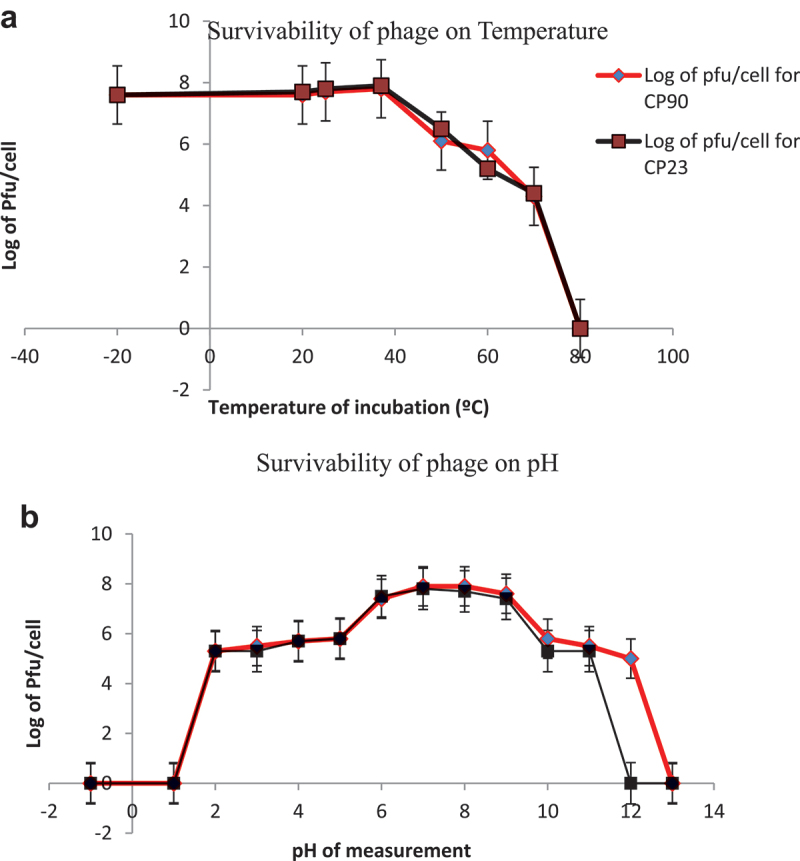
Figure 5a and b. Phage survivability studies on temperature and pH for both CP90 and CP23. Phage survivability was maintained up to 70°C with best activity below 50°C. For the pH of phage, increased phage activity was observed at an alkaline medium (6.5–9) with reduced activity below 4 to 11. Phage inactivity occurred at 1 and 13 for CP90 and 2 and 12 for CP23. Each data represents the mean value ± SD of three replicated experiments. At *p* < 0.05, there was a significant difference between CP90 and CP23 for the temperature but no significant difference at *p* < 0.05 for pH between CP90 and CP23.

The lytic phages were viable at a temperature range of −20°C to 40°C with gradual inactivation of the cells as the temperature rose to 70°C, then a complete loss of all the cells after 80°C. The CP90 showed less stability after 40°C than CP23 but the mortality rate became steady after 50°C until the sharp decline at 70°C (Figure 5a). The lytic phages showed stability within the pH of 3–11. Increased phage activity was observed in milder alkaline medium (6.5–9). Overall, there was reduced activity below pH 3 and >9 for both CP90 and CP23 (Figure 5b).

### Phage killing assay

**Figure 6.**
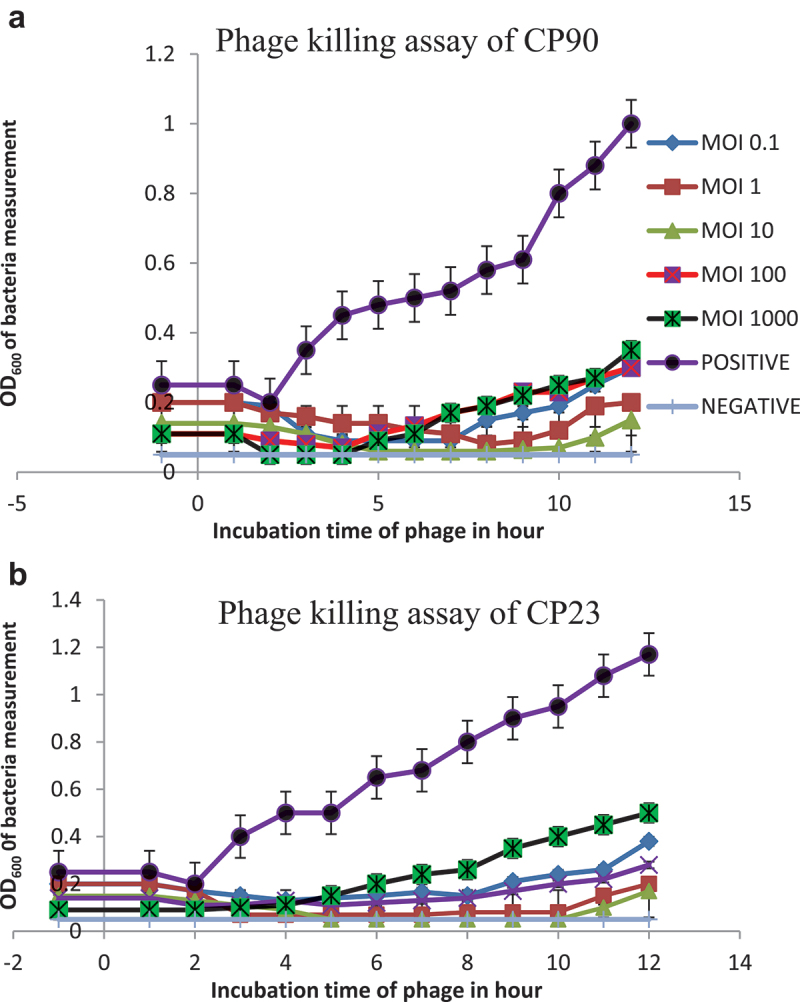
Phage killing assay for CP90; 6b: phage killing assay for CP23. The killing assay shows the *in vitro* lytic activities of the phage against the host at various MOIs of 0.1, 1, 10, 100, and 1000. All MOIs inhibited bacteria growth at 7 and 5 h post-infection treatment with the best MOI observed at 10 for CP90 and CP23 respectively. Each data represents the means ± SD of three replicate experiments.

In comparison to the unchallenged bacteria cells, the results showed that phage lysate restrained the growth at 3 h across various MOIs of post-infection, with complete inhibition maintained up to 7 and 5 h after exposure for CP90 and CP23 (Figure 6a,b). Phage treatment showed MOI-dependent response with the best growth inhibition observed at MOI 10 for CP90 and CP23.

### Frequency of appearance of bacterial mutants resistant to the phage

The frequent appearance of the CP90 mutant strain against phage lysate was 0.14% while that of CP23 was 0.2%.

## Discussion

*S. enterica* is an important zoonotic foodborne pathogen that poses a substantial threat to global health. Its primary routes of transmission include the consumption of contaminated food and water, resulting in a considerable burden of infection worldwide.^47^ The emergence of multidrug-resistant (MDR) strains of *Salmonella* due to indiscriminate use of antibiotics by humans and animals is alarmingly on the rise. The first comprehensive assessment of the global health burden caused by MDR pathogens was conducted in 2022, revealing alarming statistics. In 2019, approximately 4.95 million deaths were attributed to bacterial antimicrobial resistance, with about 1.27 million of those deaths directly caused by antibiotic abuse. This unprecedented number is expected to rise to 10 million deaths by 2050 if left unmanaged.^48–50^ The Multidrug Resistance Index (MARI) has emerged as a crucial tool for assessing bacterial resistance and its associated risk factors. A MARI value greater than 0.2 indicates that the bacteria originate from a high-risk source of contamination.^49^ Our studies have revealed that the biochemically and molecularly identified multidrug-resistant Salmonella strains isolated from our environment exhibited MARI values of 0.4 (CP90) and 0.6 (CP23) respectively. These findings suggest that these MDR strains pose a significant public health risk, highlighting the need for alternative antibacterial measures. As MDR pathogens continue to emerge, causing significant economic losses, phage therapy has become a promising alternative to traditional antibiotic treatment. Lytic phages, in particular, offer several advantages over temperate phages.^51,52^ In this study, a lytic phage and its hosts were isolated from environmental wastewater. The phage’s physicochemical characteristics, including its survivability was evaluated. Phages are ubiquitous in the environment where their host strains are. Lytic phage, like every virus is composed of protein and nucleic acid, and the morphology can be affected by temperature and pH necessitating the evaluation of its physicochemical characteristics. Phage survivability can predict bacteriophage host susceptibility and infectivity, viability, incidence of infection, and storage of phage-formulated products.^53^ In this study, the phage was found to be stable with increased activity below 50°C and then completely inactivated at 80°C. This is significant for the tropics where the temperature fluctuates between 30°C and 50°C and this invariably means that the isolated phage would persist in the environment for longer periods, increasing its potential to infect and lyse bacteria. It would also maintain its structural integrity and infectivity which is essential for phage-based applications. This is similar to other research conducted in which significant reductions of phages were observed at 50°C, thermal resilience below 70°C, and inactivation at 80 °C^11,29,54^ but not according to^39^ for whom inactivation of *Salmonella* phage was at 60°C. The phage tolerance at extreme acidic conditions indicates its ability to withstand the acidity of the stomach and thus can be administered orally. The phage stability at varied temperatures also allows its easy transportation and long-term storage without a significant change in titer. The replication pattern shows that more than 85% of the phages were adsorbed within 12 min. The latent period determined from the one-step growth curve in Figure 4, was approximately 30 min, during which about 55 new viral particles were formed. Possible variation in latent period and burst size of *Salmonella* phages may result from differences in medium, pH, temperature, or host cell.^55^ Report for Salmonella phages in other works showed long latent period and small burst size (40 min, 22 Pfu/cell, 60 min, 14 Pfu/cell), short latent period and large burst size of 20 min, 138 Pfu/cell.^56–58^ Phages with quick adsorption, short latent period and large burst possesses good characteristics for phage therapy formulation.^59^ Determining the optimal MOI is of great necessity for preparation/formulation of phage therapeutic dose and also for the sustainability of phage. In this study, it was observed that phage-resistant mutant bacteria developed rapidly with heavy phage and bacteria load (MOI 1000, 100, 0.01 and 0.1). Host inactivation occurred earlier at 100 and 1000 than the rest. The optimal MOI was determined to be 10, which was used in subsequent experiments. As observed, heavy phage and bacteria loads were found to contribute to the emergence of bacterial resistance. This suggests that heavy phage and bacteria loads perhaps is a selection pressure and should be taken into consideration in phage therapy as reported by Castillo et al.^60,61^ The frequency of mutant resistant host tested against the phage showed that CP23 developed high mutant variants compared to CP90. The ability of phages to evolve and counter the effects of rapidly mutating pathogens is a huge advantage to phage therapy. It is expected that mutant phage exists for many bacterial pathogens. Phage cocktailing and rotations can also limit the formation of mutant hosts against phages.^62–65^ The Statistical analysis revealed significant differences between CP90 and CP23 for the one-step growth curve, adsorption curve, and phage survivability at different temperatures. However, no significant difference was observed for phage survivability at different pH levels.

## Conclusion and future work

The increasing incidence and emergence of multidrug-resistant (MDR) Salmonella strains pose a significant threat to public health. Bacteriophages offer a promising bio-control solution against salmonellosis in various settings, including environmental waste management, ready-to-eat food, livestock and crop farming.

This study investigated the antimicrobial potential of a lytic phage isolated from environmental wastewater against two strains of *Salmonella enterica* obtained from sewage and abattoir wastewater in Eastern Nigeria. The characterized phage exhibited desirable features, including a short latent period, large burst size, stability across various pH conditions, and resilience to thermal stress. These characteristics suggest that this phage is a promising bio-control measure against *Salmonella enterica*.

However, further research is recommended to fully elucidate the phage’s genomic composition and classification. Additionally, animal studies are necessary to explore the therapeutic applications of this phage.
